# Veterinary drug residues in animal-derived foods: From analytical detection to intelligent and sustainable surveillance

**DOI:** 10.1016/j.fochx.2026.104643

**Published:** 2026-10-16

**Authors:** Marzieh Ramezani Farani, Danial Mirzaee, Anahita Voosough, Afrooz Hatami, Kong Chanmey, Seyed-Hassan Miraei Ashtiani, Aliya Sheik, Iraj Alipourfard, Alexandros Ch. Stratakos, Sergey A. Piletsky, Seung-Kyu Hwang, Yun Suk Huh

**Affiliations:** a Department of Biological Sciences and Bioengineering, NanoBio High-Tech Materials Research Center, Inha University, Incheon, Republic of Korea; b Department of Biology, Faculty of Science, University of Tehran, Tehran, Iran; c Department of Animal Science and Aquaculture, Dalhousie University, Truro, Nova Scotia, Canada; d Department of Pharmacotherapy and Pharmaceutical Care, Faculty of Pharmacy, Medical University of Warsaw, Warsaw, Poland; e Centre for Sustainable Agri-Food and Environment, School of Applied Sciences, University of the West of England, Bristol, Bristol, United Kingdom; f School of Chemistry, College of Science and Engineering, University of Leicester, Leicester, United Kingdom

**Keywords:** Veterinary drug residues, Food safety surveillance, Biosensors, Artificial intelligence, IoT-enabled traceability

## Abstract

Veterinary drug residues in animal-derived foods remain a major challenge for food safety, public health, and international trade. This review examines residue control through the complementary roles of analytical technologies, digital tools and green analytical chemistry. Rapid screening can support local decisions, while liquid chromatography-tandem mass spectrometry provides laboratory confirmation. Digital tools offer opportunities to connect analytical results with treatment records and product traceability, but their value depends on data quality, interoperability and validation. Solvent reduction and miniaturization should be assessed alongside energy use and material life cycles. The proposed farm-to-fork roadmap links these approaches with regulatory requirements and One Health priorities, emphasizing reliable measurements, coordinated data use and feasibility in resource-constrained settings.

## Highlights

• Veterinary drug residues pose toxicological risks across One Health domains.
• Rapid biosensors and nanomaterial platforms complement confirmatory LC-MS/MS.
• AI and machine learning support residue detection and predictive risk modelling.
• IoT and blockchain support real-time monitoring and tamper-resistant traceability.
• Green solvents and miniaturization can reduce analytical environmental footprints.

## 1 Introduction

The intensification of livestock and aquaculture production has been accompanied by extensive use of veterinary pharmaceuticals for disease control, metaphylaxis and productivity enhancement. Improper use, insufficient withdrawal times or inadequate regulatory compliance can result in drug residues in meat, milk, eggs, honey and fishery products (Atta et al., 2022; Khalifa et al., 2024; Mesfin et al., 2024; Wu et al., 2023). Acute and chronic exposure to residues above permitted limits has been linked to allergic reactions, disruption of intestinal microbiota, genotoxic and carcinogenic effects and endocrine perturbation (Khalifa et al., 2024; Rana et al., 2019). Of particular concern is the contribution of non-prudent antimicrobial use in food-producing animals to the emergence and spread of antimicrobial resistance (AMR) throughout the food chain (Bacanlı, 2024; Bacanlı & Başaran, 2019). The central challenge is to connect reliable residue measurements with timely decisions across the food chain. This review examines how analytical methods, digital tools and green analytical chemistry can be combined to support farm-to-fork residue control and identify barriers to their implementation.

Maximum residue limits (MRLs), withdrawal periods and authorization procedures provide the regulatory basis for controlling veterinary drug residues (Atta et al., 2022; Wu et al., 2023). Despite this progress, heterogeneity in MRLs across markets, variable analytical capabilities and uneven implementation of surveillance programs complicate risk management and can lead to trade disputes when consignments are above importing-country limits. These asymmetries in regulation and analysis highlight the importance of harmonized and science-based residue standards with robust monitoring systems and transparent data sharing (Atta et al., 2022; Masiá et al., 2016; Wu et al., 2023). Liquid chromatography coupled to tandem or high-resolution mass spectrometry (LC-MS/MS, HRMS) supports confirmatory analysis of structurally diverse compounds at low concentrations in complex animal matrices (Masiá et al., 2016; Pratiwi et al., 2023). These methods are usually preceded by labor-intensive steps of sample preparation. Alongside chromatographic approaches, immunoassays, biosensors and spectroscopic techniques have been developed for screening applications for which the turnaround time is faster at the cost of narrowness of analyte scope or lack of quantitative accuracy. Recent reviews have drawn attention to still existing methodological challenges such as coverage of multi-residues, harmonization of validation criteria and robust quantification in difficult matrices, recommending further levels of automation, miniaturization and integration of orthogonal detection strategies (Masiá et al., 2016; Pratiwi et al., 2023; Wu et al., 2023).

Concurrently, the food-safety domain is experiencing a fast transition to digitalization with increasing integration of artificial intelligence (AI), machine learning (ML) and data-driven analytics into contaminant-detection pipelines. AI-assisted chemometrics and deep learning (DL) models have shown better performance in processing spectral, chromatographic, and imaging data for classification, quantification, and anomaly detection, such as pesticide and veterinary drug residues (Chhetri, 2024; Lee & Kim, 2025; Liu et al., 2025; Zhang et al., 2025). At the systems level, integration of AI with Internet-of-Things (IoT) sensor networks and blockchain-based ledgers is being explored as a way to accomplish real-time monitoring, tamper-resistant traceability, and predictive risk assessment throughout distributed supply chains (Ellahi, Wood and Bekhit, 2023; Liu et al., 2025; Sobhan et al., 2025). However, the practical implementation of the technology is limited by the quality of data, interoperability, cybersecurity, and regulatory acceptance of algorithm-assisted decision-making (Chhetri, 2024; Liu et al., 2025; Zhang et al., 2025).

The environmental and resource demands of residue analysis have encouraged the adoption of green analytical chemistry (GAC) in method design and validation. Conventional LC-MS/MS techniques are often based on the use of large amounts of organic solvents, energy-consuming equipment, and disposable materials and raise concerns about occupational hazards, waste production, and life-cycle impacts. GAC concepts focus on solvent and reagent minimization, substitution by safer alternatives, miniaturization and in situ analyses, and holistic evaluation of methods by application of green metrics and life-cycle approaches (Meher & Zarouri, 2025; Mehta et al., 2024; Wu et al., 2023). In the context of residues of veterinary drugs, such green innovations are especially pertinent in the context of scaling up multi-residue monitoring and deploying sensor networks at the farm, processing and retail level by both laboratories and regulators (Mehta et al., 2024; Pratiwi et al., 2023; Sobhan et al., 2025; Wu et al., 2023).

Existing reviews examine veterinary residue detection (Pratiwi et al., 2023; Wu et al., 2023), while broader food-safety reviews address the integration of AI, IoT and blockchain for traceability (Liu et al., 2025). Building on this literature, the present review connects residue-specific screening and confirmation with traceable compliance decisions and whole-workflow environmental assessment. Its contribution is a phased implementation framework that considers how analytical errors affect downstream decisions, when digital tools add environmental burdens, and how deployment can be adapted to resource-constrained settings. The farm-to-fork sequence comprises prevention during treatment and withdrawal, screening at collection or slaughter, laboratory confirmation, and traceable product control through processing, distribution and retail. Sections 2–5 examine the supporting requirements and technologies, while Section 6.4 proposes phased implementation.

## 2 Veterinary drugs, residues and regulatory essentials

### 2.1 Veterinary drug classes and patterns

There is a wide variety of veterinary medicinal products used in food-producing species, ranging from antimicrobials and antiparasitics to anti-inflammatory or analgesic agents, hormonal and reproductive modifiers, beta-adrenergic agonists, sedatives, and anesthetics (Fig. 1) (Atta et al., 2022; Gupta, 2019; Wu et al., 2023). The use of antimicrobials and antiparasitics is the most regulated due to their high frequency of use, the narrow therapeutic index of some of these drugs, and their linkage with AMR (Adegbeye et al., 2024; Khalifa et al., 2024). Some markets and historical residue datasets are still relevant to the use of hormonal growth promoters and beta-agonists, which have been banned or strictly regulated in many countries (Adegbeye et al., 2024; Gupta, 2019). There are also variations in the use of these products among species, production systems and regions: intensive poultry and swine systems have strong reliance on group treatments for enteric and respiratory diseases, cattle and small ruminants receive injectable antimicrobials and reproductive hormones, and aquaculture systems employ bath and in-feed treatments (Adegbeye et al., 2024; Atta et al., 2022; Landers et al., 2012). A 2025 modelling study estimated global livestock antimicrobial use at approximately 110,777 t in 2019 and projected a 29.5% increase by 2040 under a business-as-usual scenario (Acosta et al., 2025).

**Fig. 1:**
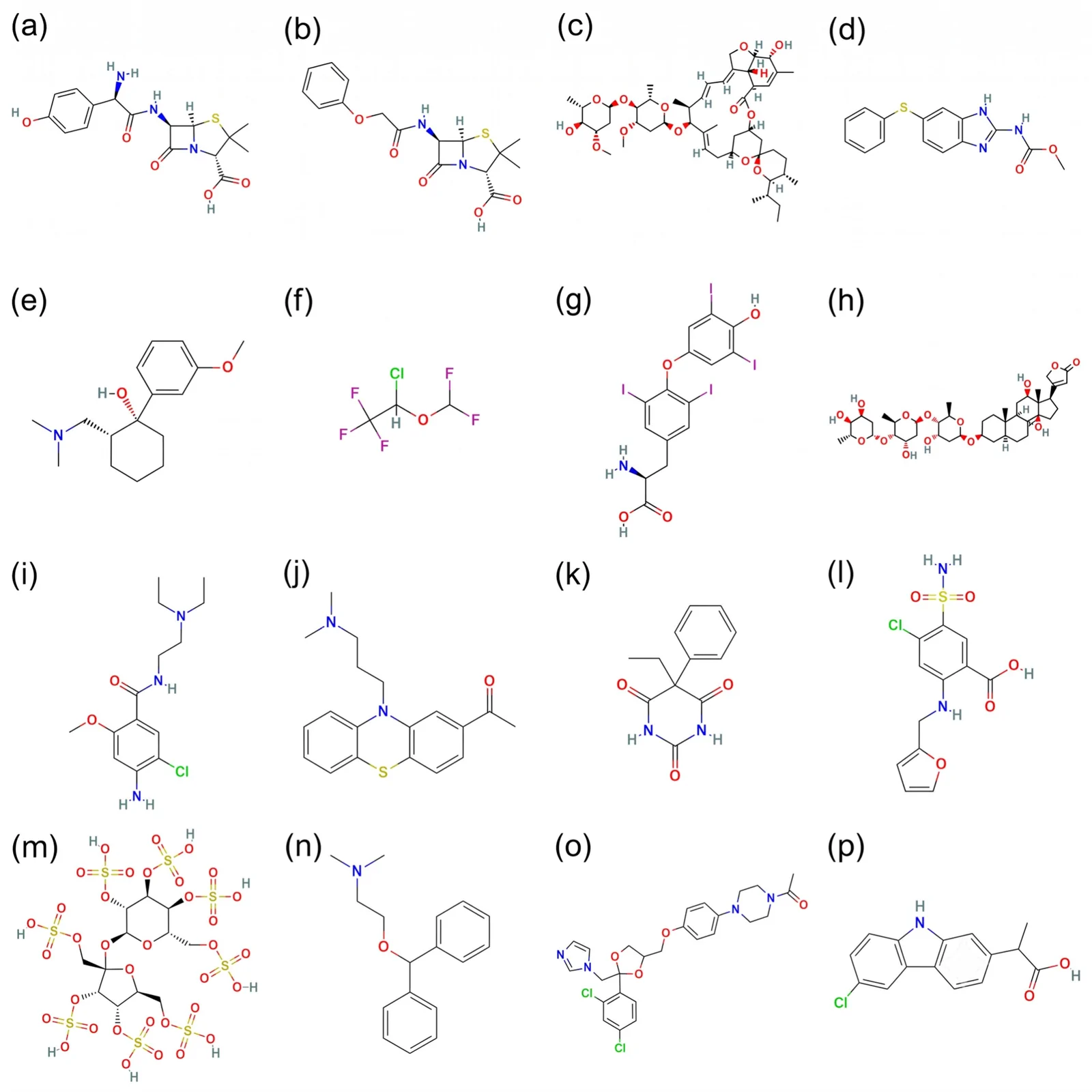
The molecular structure of several veterinary drugs: (a) Amoxicillin, and (b) Penicillin V - two common antibiotic drugs; (c) Ivermectin, and (d) Fenbendazole, are two common antiparasitic drugs; (e) Tramadol, a narcotic analgesic drug; (f) Isoflurane, an anesthetic drug; (g) Levothyroxine, a hormonal drug; (h) Digoxin, a cardiac drug; (i) Metoclopramide, an antiemetic; (j) Acepromazine, a sedative; (k) Phenobarbital, an antiepileptic; (l) Furosemide, a diuretic drug; (m) Sucralfate, a gastroprotective drug, represented by its sucrose octasulfate component in protonated form, with aluminum-containing components omitted; (n) Diphenhydramine, an antiallergic drug; (o) Ketoconazole, an antifungal drug; (p) Carprofen, an anti-inflammatory drug.

### 2.2 Residue formation and persistence

Veterinary drug residues can be parent compounds, metabolites, and associated impurities in edible tissues and animal-derived products such as meat, milk, eggs, honey, as well as aquaculture products (Atta et al., 2022; Khalifa et al., 2024; Wu et al., 2023). The formation and persistence of these depend on the absorption, distribution, metabolism, and excretion (ADME) of a drug in the target species. Lipophilic molecules like the macrocyclic lactones tend to accumulate in fatty tissues and are excreted slowly, while hydrophilic drugs like beta-lactams are excreted quickly in urine and may temporarily be found in milk (Adegbeye et al., 2024; Rana et al., 2019). Pharmacokinetics are further influenced by species, age, health, and production stage and thus these also influence the appropriate withdrawal periods (Gupta, 2019). Increasingly, physiologically based pharmacokinetic (PBPK) models are being used to estimate tissue residues after different dose regimens, which are important in making label recommendations and extra-label use decisions (Lin et al., 2025). Recent developments extend PBPK modelling into accessible, interactive decision-support tools. Zhang et al. developed an open-source model of tylosin in broiler chickens and laying hens that incorporates the reproductive system and predicts concentrations in edible tissues, egg yolk, and egg white. A web-based interface enables users to explore alternative exposure scenarios. Such tools could connect treatment records with prospective residue-depletion estimates, strengthening prevention within digital farm-to-fork systems. Their application must remain restricted to supported drug–species–regimen combinations, with model predictions distinguished from analytical verification of compliance (Zhang, Mercer, Tell and Lin, 2025).

### 2.3 Health risks associated with residue exposure

The potential health effects from residue exposure include direct toxicological and indirect routes as summarized in Fig. 2. The direct toxicities include nephrotoxicity, hepatotoxicity, neurotoxicity, carcinogenicity, reproductive effects, and endocrine-disrupting effects of hormonal agents and beta-agonists (Atta et al., 2022; Bacanlı & Başaran, 2019; Mesfin et al., 2024). Hypersensitivity reactions to drugs such as beta-lactams and sulfonamides can occur even at very low concentrations in sensitized individuals, justifying particularly stringent MRLs or prohibition of certain substances (Bacanlı & Başaran, 2019; Rana et al., 2019). At sub-inhibitory concentrations, antibiotics can favor resistant bacteria when the selective advantage exceeds the fitness cost of resistance (Gullberg et al., 2011). Resistance can involve drug inactivation, altered targets, or efflux, while mobile genetic elements enable resistance determinants to spread between bacteria. However, the contribution of dietary residues to human AMR depends on bioavailable exposure and microbial context and cannot be inferred from residue concentrations alone (Bacanlı, 2024; Khalifa et al., 2024; Lopatkin et al., 2017). Emerging evidence also suggests that chronic low-level exposure may modulate immune function and metabolic pathways, underscoring the need for precautionary management, although causal relationships remain incompletely characterized (Adegbeye et al., 2024; Bacanlı, 2024).

**Fig. 2:**
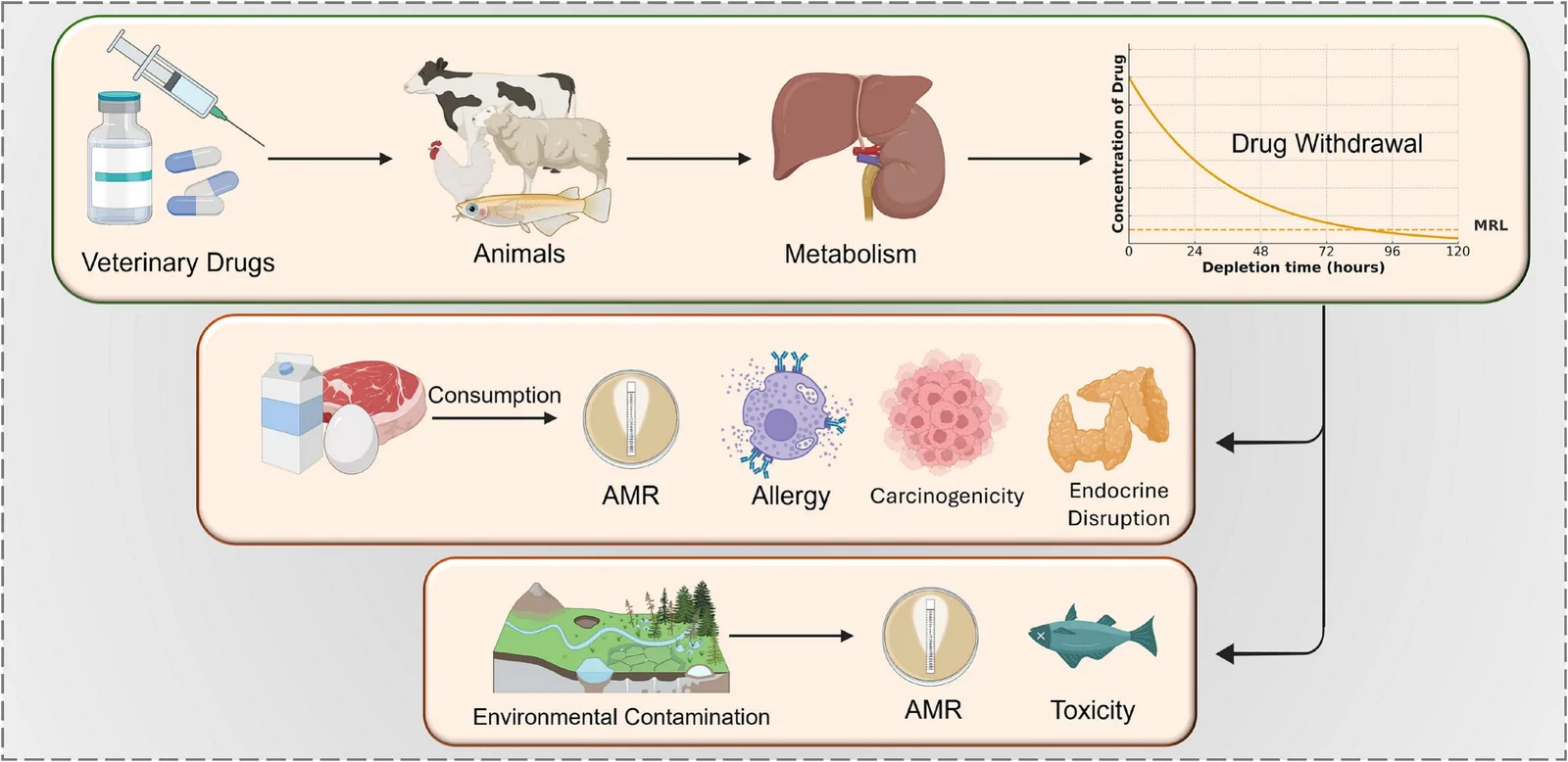
Pathways and risks of residues of veterinary drugs. Potential concerns include toxicological effects and environmental contamination; the contribution of dietary antimicrobial residues to human AMR depends on exposure and microbial context.

### 2.4 Regulatory standards and surveillance

The science-based MRLs, withdrawal periods and authorization procedures form the basis of the regulatory framework for control of residues. At the international level, the Joint FAO/WHO Expert Committee on Food Additives (JECFA) assesses toxicological, pharmacokinetic and residue-depletion data to provide recommendations for acceptable daily intakes (ADIs) and MRLs, which are then adopted or modified by the Codex Alimentarius Commission and used as global reference points in World Trade Organization dispute settlement proceedings (Atta et al., 2022; Lawrence et al., 2024; Wu et al., 2023). Although the principles are similar, the uncertainty factors and policy considerations may differ, leading to differing MRLs, which is a hurdle for international trade (Atta et al., 2022; Okunola et al., 2025). For example, Codex adopted an MRL of 10 μg/kg for ractopamine in cattle and pig muscle, whereas European Union (EU) legislation prohibits its use for growth promotion (Council of the European Union, 1996; WHO, 2012). A concrete trade example is documented in U.S. Food and Drug Administration (FDA) Import Alert 16–131: monitoring between October 2006 and May 2007 identified unapproved drug residues in 22 of 89 aquaculture samples imported from China. The alert provides for detention without physical examination of covered imports, with analytical evidence supporting shipment release (U. S. Food and Drug Administration, 2025). Compliance systems must therefore consider authorization status alongside the applicable residue definition, matrix, destination market, and standard version. Surveillance frameworks translate these standards into practice through risk-ranked monitoring plans that prioritize substances by toxicity, usage frequency, and historical non-compliance (van Asselt et al., 2023). Despite the significant progress, there are still challenges in implementing systematic enforcement, such as laboratory capacity, incomplete drug registration systems, and fragmented value chains in low- and middle-income countries, which underscore the necessity for harmonized standards, greater data transparency, and international collaboration on residue control (Adegbeye et al., 2024; Khalifa et al., 2024; Mesfin et al., 2024). These requirements provide the basis for evaluating the detection technologies discussed in Section 3.

## 3 Detection technologies: From conventional analytics to advanced sensors

Veterinary drug residue surveillance can be organized into two analytical tiers: front-line screening to identify potentially non-compliant samples, followed by confirmatory analysis to establish analyte identity and concentration. Additional wide-scope investigation is appropriate when targeted methods cannot explain suspected contamination. The assignment of a method to either tier depends on its validated performance for the intended analyte and matrix, rather than its detection principle alone (Jongedijk et al., 2023; Zhu et al., 2022).

### 3.1 Front-line screening

Microbiological inhibitor tests and enzyme-linked immunosorbent assays (ELISA) are still widely used for the first-line screening of large volumes of samples, especially for the detection of antimicrobial residues in raw milk, but suffer from cross-reactivity and limited multiplexing capabilities (Vercelli et al., 2023). Biosensors (Fig. 3a) offer rapid and potentially portable screening, but their performance depends on sample preparation, recognition selectivity, and control of matrix interference. The speed of screening technologies has improved significantly and is now a critical component between farm-level decisions and laboratory confirmation. Lateral flow immunoassays (LFIAs) (Fig. 3b) are now advanced devices that can provide semi-quantitative and even multiplex analyses and incorporate engineered labels like gold nanoparticles, quantum dots (QDs), and up-conversion phosphors (Chen et al., 2025). One of the most promising and rapidly growing fields is electrochemical biosensors (Fig. 3c), which have demonstrated sub-ng/mL detection of tetracyclines, sulfonamides, aminoglycosides, and beta-agonists in food matrices using nanomaterials like graphene, carbon nanotubes, metal nanoparticles, and metal-organic frameworks (MOFs), where electron transfer is amplified and loading of antibodies, aptamers, or molecularly imprinted polymers (MIPs) is increased (Li et al., 2026; Shen et al., 2025; Tao et al., 2020; Zhou et al., 2021). Cost and sample volume can be reduced further with screen-printed and paper-based electrochemical devices, which could be used in the field with handheld potentiostats or with smartphone interfaces (Gokdere et al., 2025). Recent developments have also combined portable electrochemical sensing with wireless smartphone-assisted readout for on-site chloramphenicol monitoring, illustrating the potential for digitally connected field screening (Dogra et al., 2025). The design space of portable and multiplexed residue screening is expanded by optical approaches such as fluorescent biosensors (Fig. 3d) based on QDs, carbon dots, and up-conversion nanoparticles (Dezhakam et al., 2024; Tao et al., 2020; Zhou et al., 2021), surface-enhanced Raman spectroscopy (SERS) (Fig. 3e) platforms that make use of drug-specific vibrational fingerprints displayed on nanostructured metal substrates (Dezhakam et al., 2024; Liu, Yang, Chen and Zhang, 2025; Qi et al., 2025), and surface plasmon resonance (SPR) (Fig. 3f) sensors that provide label-free real-time detection (Peng et al., 2020).

**Fig. 3:**
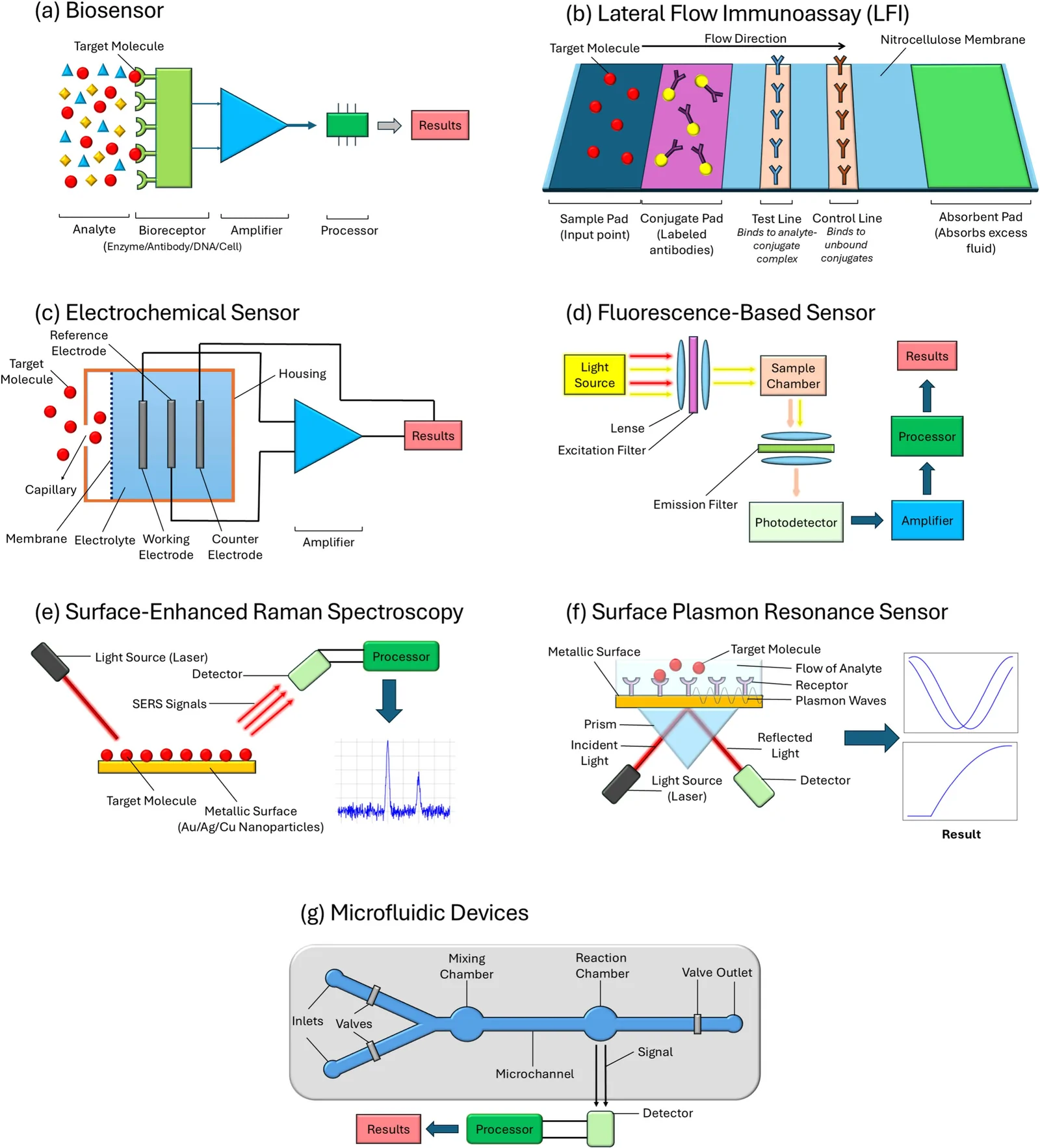
Schematic description of sensing methods for veterinary drug residue analysis. (a) Generic biosensor architecture; (b) Lateral flow immunoassay format where the sample, conjugate, test and control zones are on a nitrocellulose membrane; (c) Electrochemical sensor based on the action of working, reference and counter electrodes in order to convert binding events to electrical signals; (d) Fluorescence-based sensor with optical excitation and emission detection; (e) Surface-enhanced Raman spectroscopy (SERS) sensor where molecules of the analyte are adsorbed on a nanostructured metallic substrate; (f) Surface plasmon resonance (SPR) sensor for measurement of changes in refractive index at a functionalized metallic surface; (g) Microfluidic lab-on-a-chip device with sample manipulation and on-chip detection.

Microfluidic and multiplexed formats extend these screening approaches. Microfluidic lab-on-a-chip (LOC) (Fig. 3g) devices are small-scale systems that combine different microprocesses, such as sample pretreatment, reaction, separation, and detection, in a single package without the need for manual pipetting or handling microliter volumes, with prototype systems showing the ability to screen for antibiotic residues within 30 min (Li et al., 2026; Mun et al., 2025; Wang et al., 2022). Several drugs or drug classes can be detected simultaneously using multi-spot electrochemical arrays, multi-line LFIA strips, and microarrayed optical chips, which facilitate spatially encoded recognition elements, and replacing natural enzymes with catalytically active nanomaterials enables nanozyme-based platforms, which are more stable to temperature and pH variations in food matrices (Dezhakam et al., 2024; Li et al., 2026; Liu, Yang, Chen and Zhang, 2025; Shen et al., 2025; Zhou et al., 2021).

CRISPR-assisted biosensing provides an additional direction for small-molecule residue detection. Recognition modules can translate analyte binding into a nucleic-acid signal that activates Cas12a-mediated reporter cleavage. Zheng et al. combined upconversion nanoparticles and CRISPR/Cas12a in a near-infrared photoelectrochemical platform for quantitative kanamycin detection (Zheng et al., 2026). However, a 2026 experimental assessment found that robust Cas12a reporter detection did not consistently translate into reliable performance with previously reported small-molecule aptamers (Brandenberg et al., 2026). Residue applications therefore require independent validation of both molecular recognition and signal amplification, followed by evaluation in the intended food matrix.

### 3.2 Confirmatory analysis and wide-scope investigation

LC-MS/MS combines chromatographic separation with analyte-specific precursor and product-ion measurements for targeted identification and quantification in complex food matrices (Brandi et al., 2024). The time required for samples to be prepared has been shortened, especially with the advent of Quick Easy Cheap Effective Rugged Safe (QuEChERS) protocols and miniaturized solid-phase extraction (SPE) (Brandi et al., 2024; Zhu et al., 2025). In addition to triple quadrupole instruments, ultra-high-performance LC (UHPLC) coupled to Orbitrap or time-of-flight analyzers can be used for targeted quantification as well as suspect or non-targeted screening of full-scan data, which can identify over 200 analytes in a single run and enable retrospective analysis for emerging residues (Amelin et al., 2024; Brandi et al., 2024). Ambient ionization techniques like paper-spray mass spectrometry ionize the analytes directly from paper substrates within seconds, thereby reducing sample preparation and creating opportunities for deployment of portable or benchtop mass spectrometers at inspection points (Su et al., 2018). The use of MS alone does not establish confirmatory status; each workflow must meet the identification and validation requirements applicable to its intended use (European Commission, 2021).

### 3.3 Critical comparison and method selection

Method selection should begin with the intended surveillance decision. Front-line screening is useful at milk collection points, slaughterhouses and processing facilities where many samples require rapid assessment, whereas confirmatory analysis establishes the identity and concentration of suspected residues. The sensitivity, selectivity, cost and portability criteria summarized in Fig. 4 should therefore be considered alongside the matrix-specific evidence in Table 1. A low detection limit alone does not establish suitability for routine surveillance, particularly when studies examine different analytes, sample preparation procedures and concentration ranges. Samples associated with strong evidence of misuse, inconclusive screening results or substances outside the validated screening panel may require direct laboratory investigation (Brandi et al., 2024; Vercelli et al., 2023).

**Fig. 4:**
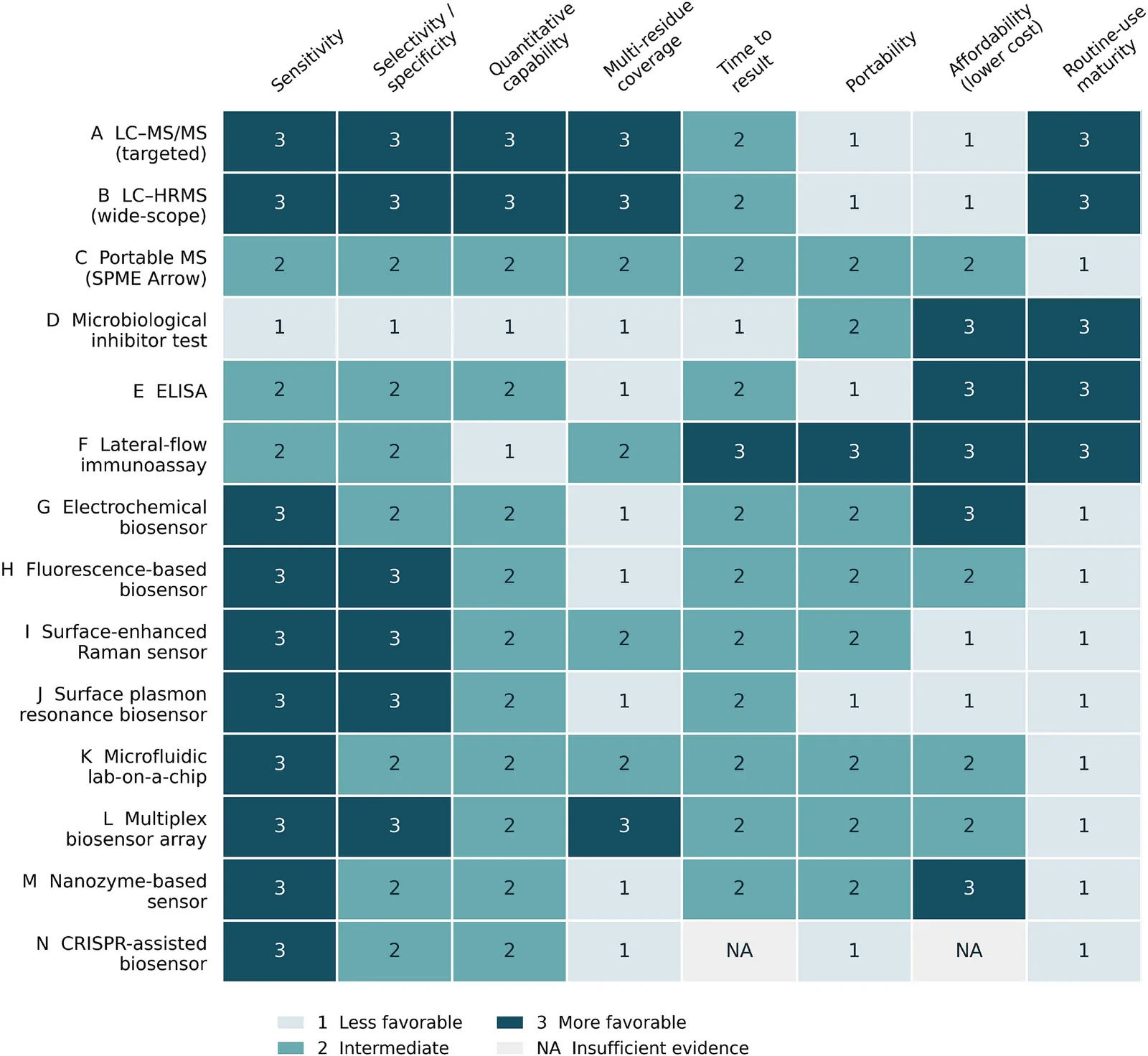
Qualitative comparison of analytical platforms for veterinary drug residue detection. Ratings reflect the authors' judgments informed by studies cited in this review: 1 = less favorable, 2 = intermediate, and 3 = more favorable. No standardized scoring instrument or fixed numerical thresholds were applied. Higher ratings indicate more favorable attributes, including shorter workflow time and lower equipment and operating costs. Ratings are ordinal, unweighted, and not combined into an overall ranking. Gray NA cells indicate insufficient evidence. Statistical uncertainty was not quantified; ratings may vary with analyte, food matrix, sample preparation, and implementation. Routine-use maturity denotes established use for the intended purpose, rather than regulatory acceptance of individual assays. Platform categories may overlap.

**Table 1:** Advancements in diagnostic tools to detect veterinary drug residues.

**Tool / Platform**	**Representative target(s)**	**Matrix**	**Transduction principle**	**Reported performance (LOD/LOQ, accuracy)**	**Time-to-result / Throughput**	**Multiplexing & POC suitability**	**Key limitations (typical)**	**Ref.**
LFIA (lateral-flow immunoassay)	Imidocarb	Milk, beef	Competitive immunochromatography (AuNP label)	Visible signal reduction at 5 ng/mL in milk and 10 ng/mL in beef; complete test-line disappearance at 20 and 50 ng/mL, respectively; qualitative or semiquantitative interpretation	5–10 min strip development after sample preparation; beef extraction requires additional time	POC-ready; amenable to multiplex lines	Semi-quantitative; matrix effects; positives need confirmatory MS	(Xu et al., 2021)
LFIA (multiplex fluorescent microsphere)	Colistin & bacitracin	Milk	Sandwich/competitive LFIA with fluorescent beads	Simultaneous screening of two peptide antibiotics with portable reader (demonstrated in milk)	∼10–15 min; portable reader	Multiplexed dual-analyte	Calibration/reader dependence; strip reproducibility	(Li et al., 2020)
LFIA (bacitracin)	Bacitracin	Milk	Competitive LFIA (AuNP)	Validated vs. ELISA in 90 milk samples; on-strip quantitation feasible	∼10 min	POC	Cross-reactivity panel required for routine use	(Byzova et al., 2020)
Electrochemical sensor (nanoporous Au-Ag/rGO/polyglycine on GCE)	Metronidazole	Foods (spiked)	Voltammetric signal change upon reduction	Ultra-trace sensitivity reported with broad linear range (device-specific); designed for residue-level MTZ	Minutes per run	Portable potentiostat capable	Electrode conditioning; potential interferents	(Adane et al., 2024)
Electrochemical sensor (F-doped activated carbon + hydrophobic DES)	Oxytetracycline	Shrimp and shrimp pond water	Differential pulse voltammetry on modified SPE	LOD 1.74 μg/L; recoveries 83.8–100.5% in shrimp pond water and 93.3–104.5% in shrimp	Minutes per run	Portable; low-cost electrodes	pH and fouling effects; matrix deconvolution	(Wannasri et al., 2022)
Aptamer fluorescence (G-quadruplex structure-switching)	Enrofloxacin	Food & water	Split-aptamer G4 switch with ThT/SG-I reporter	nM-level LOD (reported ∼26.7 nM in study) with selectivity vs. analogs	≤30 min; microplate/hand-held fluorimeter	Potential for panelization with aptamer arrays	Aptamer stability; ionic-strength sensitivity	(Shen et al., 2024)
QD-FLISA (quantum-dot fluorescence immunoassay)	Florfenicol	Animal-derived foods	QD-labeled antibody; fluorescence readout	LOD ∼0.3 ng/mL; quant. Range ∼ 0.6–30.4 ng/mL (study-specific)	≤1 h; microplate	Multiplex feasible with spectrally distinct QDs	QD handling; photobleaching; instrument need	(Xin et al., 2024)
SERS (engineered Au@Ag on supports; application example)	Nitrofurantoin (nitrofurans)	Honey (also meat/milk in related work)	Raman enhancement on nanostructured metals	Sub-mg kg^−1^ detection (study reported quantitative NFT in honey; methods achieve rapid single-scan reads)	Seconds per spectrum; high throughput scans	Amenable to multiplex via spectral unmixing	Substrate reproducibility; matrix adsorption	(Zu et al., 2025)
SPR biosensor (mAb-based)	MQCA (olaquindox marker residue)	Swine tissues (muscle, liver)	Label-free RI shift upon binding	LOD ∼1.4 μg/kg (muscle) and ∼ 2.7 μg/kg (liver); below regulatory limits used in study	Real-time; few minutes per injection	Chip-level multiplex possible	Matrix clean-up; surface regeneration	(Peng et al., 2020)
Microfluidic fluorescent platform (QD-based)	Pefloxacin, malachite green and 1-aminohydantoin hydrochloride	Prepared aqueous solutions; aquatic-product applications proposed	Fluorescence quenching of ligand-capped CdTe quantum dots in a microfluidic platform	Pefloxacin LODs of 7.57, 1.75 and 2.90 μM for MPA-, TGA- and NAC-capped CdTe probes, respectively; these values describe solution-phase probe measurements	Fluorescence response within seconds under the reported solution conditions; complete food-testing workflow not established	Three parallel probe channels; potential for visual screening	Food-matrix validation and field performance remain to be established	(Li et al., 2022)
Portable MS + SPME Arrow (ambient/mini-MS)	Amantadine, thiabendazole, sulfamethazine, clenbuterol, ractopamine	Milk, chicken	On-site MS with mesoporous-silica SPME-Arrow pre-concentration	Approximately 30 s MS measurement after 15 min extraction and 30 min desorption, excluding initial sample preparation	Seconds per analyte; field-deployable	On-site identification and quantification; regulatory confirmation requires validation against applicable criteria	Lower resolving power vs. benchtop; library coverage	(Lan et al., 2024)
MOF-mediated immunosensor (Ce/Zr-UiO-66 probe)	Enrofloxacin	Milk	Optical immunoassay using a Ce/Zr-UiO-66 signaling probe and a polydopamine-coated pipette-tip carrier	Reported detection limit: 0.12 ng/mL	<15 min reported determination time; sample-preparation time should be accounted for separately	POC-amenable with simple readout	Batch-to-batch MOF variability	(Dong et al., 2024)
HRMS non-targeted / wide-scope LC-HRMS	≥100 veterinary drugs & metabolites (example: 141 in pork)	Meat (transferable to other matrices)	Accurate-mass full-scan with data-driven suspect/NTS	Broad-scope detection with retrospective mining; isotopic features improve ID	Hours per batch; high-throughput autosampler	Lab-based; complements screening	Capital cost; data triage & validation load	(Jongedijk et al., 2023)
Targeted LC–MS/MS multi-residue	Common veterinary drug panels	Animal-derived foods	Chromatography + SRM/PRM MS/MS	Regulatory-grade sensitivity/specificity; established workflows for screening + confirmation	Batch throughput depends on extraction capacity, chromatographic run time and quality-control injections	Lab-based; gold standard confirmatory method	Solvent use; skilled staff; CAPEX/OPEX	(Brandi et al., 2024)

Reported assay or acquisition times exclude sample preparation unless explicitly stated. Throughput also depends on parallel processing, calibration, quality-control measurements and repeat analysis. Performance values are specific to the reported analyte, matrix and validation conditions.

**Abbreviations:** LOD, limit of detection; LOQ, limit of quantification; LFIA, lateral-flow immunoassay; AuNP, gold nanoparticle; GCE, glassy carbon electrode; rGO, reduced graphene oxide; DES, deep eutectic solvent; QD(s), quantum dot(s); ThT, Thioflavin-T; SG-I, SYBR Green I; SERS, surface-enhanced Raman spectroscopy; SPR, surface plasmon resonance; MQCA, 3-methyl-quinoxaline-2-carboxylic acid; QD-FLISA, quantum-dot fluorescence immunoassay; HRMS, high-resolution mass spectrometry; LC-MS/MS, liquid chromatography-tandem mass spectrometry; MS, mass spectrometry; SPE, solid-phase extraction; POC, point-of-care; MOF, metal-organic framework; MPA, 3-mercaptopropionic acid; TGA, thioglycolic acid; NAC, *N*-acetyl-L-cysteine.

Three examples from Table 1 illustrate differences in matrix handling and interference. For the imidocarb LFIA, milk required dilution, whereas beef required extraction and defatting. The antibody also showed 18.12% cross-reactivity with 4,4′-dinitrocarbanilide in ic-ELISA testing, indicating a potential selectivity limitation without establishing the false-positive rate of the finished strip (Xu et al., 2021). For the oxytetracycline electrochemical sensor, recoveries were 83.8–100.5% in shrimp pond water and 93.3–104.5% in shrimp, but related tetracyclines affected the signal. Acceptable recovery therefore does not demonstrate complete discrimination among coexisting drugs (Wannasri et al., 2022). The SPME Arrow–portable MS method supported measurement of five drugs in milk and chicken, although reported matrix effects of 51.3–69.9% required matrix-matched calibration (Lan et al., 2024). Together, these examples show that neither selective recognition nor mass-selective detection removes the need to evaluate interference in the intended matrix.

Reproducibility and stability provide a further basis for comparison. The main articles describing the selected LFIA and electrochemical sensor do not quantify manufacturing batch reproducibility or long-term storage performance. Triplicate recovery measurements in the electrochemical study establish neither characteristic. Similarly, storage of the LFIA strips at 4 °C with desiccant does not establish a validated shelf life (Wannasri et al., 2022; Xu et al., 2021). Lan et al. reported extraction relative standard deviations (RSDs) below 7.0% within batches and 9.3% between batches, together with reuse testing over 50 extraction and desorption cycles. However, these tests used standard mixtures and do not demonstrate equivalent durability in food matrices or stability during prolonged storage (Lan et al., 2024). Repeatability, batch reproducibility, reuse durability and shelf life should consequently be evaluated separately.

Operational comparisons must also include the complete workflow. LFIA and screen-printed electrodes offer relatively simple screening formats, but readers, extraction, consumables and repeat testing contribute to their practical cost. Parallel assays can increase throughput, although sample preparation may remain the limiting step. For example, the approximately 30 s measurement reported for portable MS followed 15 min extraction and 30 min desorption (Lan et al., 2024). Laboratory LC-MS/MS requires greater investment in instrumentation, maintenance and trained personnel, but provides the identification and quantification needed for confirmation. Its batch throughput depends on extraction capacity, chromatographic run time and quality-control injections. Wide-scope liquid chromatography-high-resolution mass spectrometry (LC-HRMS) extends analyte coverage and retrospective investigation, while adding requirements for data processing and verification of tentative identifications (Brandi et al., 2024; Jongedijk et al., 2023).

Decision errors should be assessed at defined concentrations and screening cutoffs, rather than inferred from detection limits, recoveries or calibration correlations. False-negative screening results may prevent contaminated samples from reaching confirmation, whereas false-positive results increase laboratory workload. As a regulatory benchmark, Commission Implementing Regulation (EU) 2021/808 requires screening methods to demonstrate a false-compliant rate of no more than 5%. For confirmatory methods, the permitted α error is no more than 1% for prohibited or unauthorized substances and 5% for authorized substances. These limits are validation requirements, not measured error rates for the platforms in Table 1. Where suitable validation data are absent, platform-specific rates remain undetermined (European Commission, 2021).

Digital and green approaches support both analytical tiers. AI can assist signal interpretation and sampling priorities, but its predictions require validation against reference measurements and representative samples. IoT can connect analytical results with treatment, location and process records, while blockchain supports an auditable history of those records without establishing the accuracy of the original measurement. Green analytical methods should be assessed against extraction efficiency, analytical reliability, energy demand and material use across the same workflow. Screening may reduce the number of samples requiring resource-intensive confirmation, but excessive false-positive referrals or repeated measurements can diminish the expected savings. The practical objective is therefore to combine reliable screening, appropriate confirmation and traceable records while reducing the total burden of surveillance (Liu et al., 2025; Nowak et al., 2023; Zhou et al., 2024).

## 4 Digital intelligence and traceable surveillance systems

Digitalization is transforming veterinary drug residue control, supporting the integration of laboratory measurements, field screening and supply-chain records. Integrating AI, IoT sensor networks, and distributed ledgers enables high-frequency data capture, automated signal interpretation, and tamper-evident compliance records, aligning residue control with Food Industry 4.0 and One Health strategies (Dakhia et al., 2025; Liberty et al., 2025; Liu et al., 2025; Pandhi et al., 2025; Wu et al., 2023). The following subsections distinguish three complementary functions: AI interprets analytical data, IoT connects measurements and contextual records, and blockchain can provide a shared audit trail. Their integration depends on a common sample or batch identity and explicit rules for acting on screening and confirmatory results.

### 4.1 AI and machine learning in residue detection pipelines

AI and ML are increasingly behind analytical pipelines for veterinary drug residues through automated feature extraction, pattern recognition, and decision support from high-dimensional chromatographic, spectral, and imaging data. Rather than using single peak integration and univariate thresholds, multivariate ML models use subtle relationships between instrument responses and residue concentrations, enhancing sensitivity and robustness in complex matrices such as meat, milk, and eggs (Pandhi et al., 2025; Wu et al., 2023). Recent extensive reviews on the use of AI in food safety include applications across chemometric pre-processing, non-target screening, automated quality classification, and anomaly detection of large routine data sets (Dhal & Kar, 2025; Pandhi et al., 2025). DL architectures have demonstrated high performance in the processing of hyperspectral images, Raman spectra, and the output of sensor arrays for non-destructive screening of contaminants and residues at line speed. Coupled with cloud infrastructures or embedded accelerators, such models can be deployed in the proximity of production environments and transform residue analysis from purely confirmatory testing to an intelligent and predictive layer within hazard-control systems (Dhal & Kar, 2025; Liu et al., 2025; Sonwani et al., 2022).

Representative AI/ML applications spanning embedded immunoassay readers, hyperspectral imaging classifiers and ML-assisted SERS analysis are summarized in Table 2, illustrating the breadth of opportunities for AI to augment both field-level screening and regulatory risk modelling. Despite these advances, AI integration into residue pipelines raises concerns around data quality, model generalizability, and regulatory acceptance. Explainability tools such as SHapley Additive exPlanations (SHAP), applied to hyperspectral models for ofloxacin residues, can identify spectral regions influencing model predictions, although feature attribution alone does not establish chemical specificity or transferability (Dong et al., 2025). Standardized validation protocols and lifecycle management procedures will be essential before such tools can be safely embedded in official control workflows (Dhal & Kar, 2025; Liberty et al., 2025; Pandhi et al., 2025). SHAP assigns contributions to individual features relative to a background prediction, whereas local interpretable model-agnostic explanations (LIME) approximate model behaviour around an individual prediction using an interpretable local model (Lundberg & Lee, 2017; Ribeiro et al., 2016). For residue screening, these explanations should be checked against plausible spectral bands, assay regions and known matrix interference (European Commission, 2021). Agreement with chemical expectations is useful, but does not prove that a model detects the residue itself. Correlated wavelengths and the choice of background or perturbation samples can affect interpretation (Aas et al., 2021). Explanation stability should therefore be examined across independent samples, instruments and production batches.

**Table 2:** Selected AI/ML applications in veterinary drug residue analysis and related assay-readout research. Performance values describe the individual study conditions and are not directly comparable. Object-detection mAP, classification accuracy and concentration-prediction R^2^ assess different outcomes and do not establish regulatory screening performance on their own.

Tool / Platform (AI/ML)	Representative target(s)	Matrix	AI/ML method used	Reported performance / outcome	Time-to-result / Throughput	POC suitability	Notes/limitations	Ref.
Embedded reader for CGIA/LFIA test cards	Sulfonamides, trimethoprim, florfenicol (test cards)	Animal urine; food-matrix transfer requires separate validation	Lightweight object detection (VetStar) with knowledge distillation on RK3568	mAP50 97.4; mAP50–95 89.5; ∼5.4 s inference per run on-device	Seconds per test; high-throughput tray	Embedded on-device analysis; field ruggedness requires validation	Image-based interpretation improves consistency vs. visual reads; still screening (not confirmatory)	(Li et al., 2025)
Smartphone-image classification of LFA analyte-load categories	SARS-CoV-2 nucleocapsid protein	Recombinant protein in extraction buffer; images of COVID-19 antigen test strips	Random forest and CNN models for classification of analyte-load categories	Random forest and CNN models classified protein-load categories; random forest performed better on small images, whereas CNN models were more robust to image noise	15 min assay development, followed by image capture and computational analysis	Smartphone-based readout with controlled lighting	Methodological example outside veterinary-residue analysis; transfer to antibiotic LFAs requires assay-specific training and independent validation	(Davis & Tomitaka, 2025)
HSI + explainable AI for VDR in mutton	Ofloxacin (example)	Mutton	CNN-SSAE with SHAP-based wavelength attribution	Integrated qualitative/quantitative detection demonstrated (feature wavelength explainability reported)	Rapid imaging; batch scans	Moderate (lab-portable HSI rigs exist)	Ofloxacin in mutton; transfer to other drugs, matrices and instruments requires validation	(Dong et al., 2025)
HSI + DL for multi-drug residues in beef	Metronidazole, ofloxacin, salbutamol, dexamethasone	Beef	CNN with DWT/CARS/PCA feature reduction	DL + DWT improved classification speed/accuracy for residue recognition (non-destructive)	Rapid imaging; sample-to-decision in minutes	Moderate	Requires spectral libraries; performance matrix-dependent	(Jiang et al., 2022)
SERS + self-optimizing ML quantification	Ciprofloxacin, norfloxacin	Milk	Self-optimizing ML on SERS spectra	R^2^ up to 0.996 for predicted vs. actual concentration	Seconds per spectrum	Good (portable Raman)	Substrate/matrix effects; needs robust calibration	(Liu, Xu, Fang, Li and Hasi, 2025)
SERS (COF-Ag substrate) + SVM	Multiple antibiotics (structurally similar)	Spiked food matrices (study)	PCA for DR + SVM classification	100% classification accuracy among antibiotics (spectral discrimination)	Seconds per spectrum	Good	Performance may vary on real-world matrices	(Shang et al., 2025)
1-D CNN-assisted SERS for multi-sulfonamides	Sulfonamide class	Aquatic environments; food-matrix validation is not established by this study	1-D CNN on SERS spectra	Deep-learning discrimination of multi-sulfonamides demonstrated	Seconds per spectrum	Good	Requires trained models per substrate	(Ji et al., 2023)

**Abbreviations:** AI, artificial intelligence; ML, machine learning; DL, deep learning; CGIA, colloidal gold immunoassay; LFIA/LFA, lateral-flow (immuno)assay; mAP, mean average precision; HSI, hyperspectral imaging; CNN, convolutional neural network; SSAE, stacked sparse autoencoder; DWT, discrete wavelet transform; CARS, competitive adaptive reweighted sampling; PCA, principal component analysis; DR, dimensionality reduction; SERS, surface-enhanced Raman spectroscopy; SVM, support vector machine; RT, retention time; LC-HRMS (or LC/HRMS), liquid chromatography-high-resolution mass spectrometry; NTS, non-target screening; POC, point-of-care.

Generalizability should be assessed using samples from farms, production batches, instruments and sampling periods excluded from model development. Spectra or images from the same biological sample must remain in the same data partition, and preprocessing and feature selection should be fitted within the training folds (Vabalas et al., 2019). Evaluation should include matrix variation, mixtures, concentrations around the screening decision level and, where available, incurred residues. Relevant outcomes include sensitivity, specificity, false-negative rates and prediction error near the decision level, with uncertainty intervals. A high classification accuracy or correlation within one dataset does not establish reliable performance at a new site. Samples outside the validated domain should be flagged for further testing.

Few-shot learning could help when labeled examples of a new residue class are scarce. Prototypical networks learn an embedding in which a small support set defines each class prototype and query samples are classified by their distance from these prototypes (Snell et al., 2017). For residue analysis, this strategy could be investigated for spectral classification, but adaptation to a new instrument or food matrix also requires explicit testing for domain shift. Benchmarking should compare few-shot models with conventional chemometric models and transfer learning under the same sample budget. Few-shot classification does not by itself establish quantitative calibration or reliable decisions near an MRL. It is therefore proposed here as a research direction.

Self-supervised foundation models offer a complementary strategy for learning from large collections of unannotated analytical data. DreaMS, first published online in 2025, learns molecular representations from tandem mass spectra and can be fine-tuned for spectral similarity, molecular fingerprint and chemical-property prediction. For veterinary-residue surveillance, this approach could support prioritization of unfamiliar signals and structurally related metabolites in LC-HRMS investigations. This is a proposed application: the published results do not establish regulatory performance for veterinary residues. Evaluation should address independent food matrices, instrument variation and erroneous annotations, with candidate identities verified using appropriate analytical evidence (Bushuiev et al., 2026).

### 4.2 IoT-enabled sensor networks and edge analytics

IoT technologies go beyond isolated laboratory residue measurements by networking distributed sensors that capture environmental, process and, in some cases, direct residue-related signals across the food chain. Real-time biosensing-based active or intelligent packaging techniques can track signs of spoilage, migration or contaminant build-up in meat and dairy products and send data wirelessly to quality-control platforms (Sobhan et al., 2025). Comprehensive bibliometric analyses indicate a swift growth in the application of IoT in food safety, including temperature-humidity recording, gas sensing, and location tracking for cold-chain integrity, which describe cold-chain conditions but do not directly measure veterinary drug residues (Bouzembrak et al., 2019). In the agricultural and food processing industries, IoT architectures are combined with big data analytics and AI to support continuous surveillance of critical control points to allow for early detection of deviations that may pose an increased risk of developing violative residues or co-occurring hazards (Misra et al., 2022). These developments redefine residue control as part of an extensive cyber-physical system in which process variables, product properties, and analytical signals are optimized together to ensure safety and quality.

Residue-specific examples distinguish local digital readout from cloud-connected analysis. Lu et al. developed LumiCellSense, a smartphone-based system combining a 16-well chip containing bioluminescent bacterial reporters with image processing and automatic alerts. The device detected ciprofloxacin in spiked whole milk with a reported detection threshold of 7.2 ng/mL. This demonstrates local sensing and computation; networked surveillance was not demonstrated. The biological response time and need to maintain reporter activity also distinguish the platform from continuously operating environmental sensors (Lu et al., 2019).

A more direct example of connected residue analysis is the ZnMOF-74 and Mn:ZnS quantum-dot sensor array reported by Deng et al., which used a smartphone application with cloud-based machine learning. The study examined enrofloxacin, chlortetracycline, oxytetracycline and tetracycline in milk, honey and beef, reporting 98% recognition accuracy and recoveries of 97.33% to 107.40%. This links residue-responsive sensing with remote computation, but does not establish an autonomous, continuously operating farm-to-fork network. Deployment would additionally require traceable sample identification, stable calibration, assay handling and evaluation of connectivity failures (Deng et al., 2026).

Low-cost IoT nodes measuring temperature, humidity, and volatiles, combined with on-device classification models, can identify spoilage-related changes, but cannot establish veterinary residue concentrations without a validated residue-specific measurement (Popa et al., 2019; Sonwani et al., 2022). In a proposed edge-cloud workflow for residue control, the reader or gateway would verify sample identity, check assay or image quality, run a validated lightweight model and retain a time-stamped local result. Cloud services would combine records from multiple sites with laboratory results and treatment histories, assess drift and manage model versions. Local processing can maintain a provisional screening service during connectivity loss, whereas centralized analysis supports broader surveillance and controlled model updating (Beri & Sachdeva, 2026; Dakhia et al., 2025). At a milk collection point, for example, a connected test reader could retain the batch identifier, assay lot, model version, screening result and quality-control status locally, then synchronize the record when connectivity returns. Suspect results would prompt segregation and confirmatory testing under an agreed protocol. Failed quality checks or predictions outside the validated domain would trigger an indeterminate result rather than automatic release. Cloud updates should be validated before deployment, signed and versioned, with rollback available (Fagan et al., 2020). Duplicate or delayed uploads should be reconciled without changing the original measurement time. This workflow remains a proposed implementation scenario. For veterinary residues, integrating sensor metadata with laboratory results could support dynamic sampling plans and residue-depletion prediction (Beri & Sachdeva, 2026; Dakhia et al., 2025; Liu et al., 2025; Misra et al., 2022; Popa et al., 2019). Edge processing limits transmission of raw data and dependence on connectivity, but constrains memory, model size and local maintenance. Cloud processing offers greater computational capacity and cross-site analysis, but adds transmission delays, service costs and data-governance requirements. Evaluation should separate assay time, local inference, upload and database commit time, and report energy per test, data volume, outage recovery and false-negative performance. Rapid inference cannot compensate for slow sample preparation or an unreliable assay.

However, IoT-enabled residue surveillance must overcome some practical limitations in the areas of connectivity, cybersecurity, and sustainability. Reviews point to pervasive challenges including heterogeneous communication standards, lack of semantic interoperability between devices and databases, and vulnerabilities to tampering or spoofing that could compromise data integrity (Bouzembrak et al., 2019; Liberty et al., 2025). Energy consumption and electronic waste related to dense sensor deployments are also pressing issues, especially when continuous monitoring of temperature is needed in refrigerated warehouses, transport vehicles, and retail cabinets (Bouzembrak et al., 2019; Misra et al., 2022; Sobhan et al., 2025). From a residue control perspective, it is critical that sensor networks are risk-based and targeted; otherwise, they may produce high-volume but low-value data that overload analytical capacity and fail to enhance decision-making (Liberty et al., 2025; Wu et al., 2023). Emerging best practice therefore emphasizes modular architectures, secure communication protocols, and prioritization of data streams that inform exposure assessment, withdrawal times, and compliance verification, either directly or indirectly (Beri & Sachdeva, 2026; Bouzembrak et al., 2019; Misra et al., 2022).

### 4.3 Blockchain-supported traceability and digital records

Blockchain technologies provide a complementary layer for ensuring and sharing information related to residues in complex chains of food of animal origin. Systematic reviews of blockchain in the agri-food sector report on a range of pilots and implementations of blockchain in meat, dairy and seafood where distributed ledgers are used to record farm inputs, treatments, logistics events and quality certificates as time-stamped records whose subsequent alteration can be detected (Antonucci et al., 2019). Such systems can substantially reduce the time needed to identify implicated batches of products in cases of contamination and increase transparency for downstream buyers and consumers (Rajput et al., 2025; Stranieri et al., 2021). Firm-level studies have shown that early adopters of blockchain-based traceability tend to be larger agri-food companies but also demonstrate how these technologies can help improve supply chain performance and trust when paired with the right governance and data-sharing agreements (Giannini et al., 2025; Stranieri et al., 2021). Although the majority of case studies currently being developed have an emphasis on authenticity and microbiological hazards, the same infrastructure can be used to store the results of laboratory residue testing, treatment histories and withdrawal compliance records that can be made immediately available to regulators and to trading partners (Antonucci et al., 2019; Giannini et al., 2025; Rajput et al., 2025; Stranieri et al., 2021).

A practical residue-control design could use a permissioned network with authenticated laboratories, processors and regulators. Large analytical files and sensitive treatment records would remain in controlled repositories; shared transactions would contain batch identifiers, result status, timestamps, and cryptographic hashes linking them to the source records. An authorized laboratory interface would submit signed results, and smart contracts would check the submitter and applicable batch rules before committing a status change. Hyperledger Fabric also supports private data collections, in which selected participants retain the data while the shared ledger records a hash (Hyperledger Fabric, n.d). Neither a hash nor network consensus establishes that a sample was representative or an assay was correct.

Beyond simple record keeping, blockchain can be used to support programmable logic in the form of smart contracts that can automate aspects of residue compliance. For example, systems have been proposed in which laboratory- or sensor-generated quality metrics are written to a ledger and assessed by smart contracts which cause a payment, an alert or logistic action to be taken only when predefined safety criteria are satisfied (Yu et al., 2020). Alzahrani et al. provide a more explicit recall-logic example in a Hyperledger Fabric simulation using 190 food-related records. Their smart contracts evaluate batch inconsistencies and fraud indicators, change the status of flagged batches and issue stakeholder notifications. This illustrates executable recall initiation, but the evaluation used simulated events without physical IoT sensors and did not demonstrate a veterinary-residue recall in commercial operation. Transfer to residue control would require authenticated analytical evidence and substance- and matrix-specific decision rules (Alzahrani, Kostkova and Alshammari, 2026). Business and management studies emphasize the need for effective integration of blockchain in food supply chains to be aligned with existing enterprise resource planning systems, clear models for the ownership of data, and mechanisms for linking digital records to physical products (e.g., through the use of identification systems such as radio-frequency identification (RFID) or a QR code) (Cozzio et al., 2023; Stranieri et al., 2021).

For a milk batch, a proposed contract could distinguish pending, held, released and recalled states. A suspect screen would change the batch to held and request confirmatory analysis. An authorized laboratory result would then be evaluated against the applicable residue definition and decision rule, including measurement uncertainty where required. Confirmed non-compliance could trigger a distribution block and initiate notifications for linked downstream lots under an agreed recall procedure. Physical segregation, retrieval and verification of recall effectiveness would remain operational responsibilities. The trigger, responsible authority and subsequent actions should be recorded explicitly, and any correction should be linked to the original event. Blockchain is most useful where several organizations need a jointly auditable record and do not rely on a single data custodian. Where a trusted custodian already exists, a controlled database with signed records and an audit trail may be simpler to operate (Wüst & Gervais, 2018). The choice should therefore be justified by governance requirements, transaction costs and maintenance capacity, rather than by traceability alone.

### 4.4 Toward integrated smart residue-control architectures

AI, IoT and blockchain can support the screening and confirmatory tiers described in Section 3 by connecting analytical results with treatment records, sampling priorities and compliance actions. In such systems, validated screening devices provide measurements at defined sampling points, edge models assist local interpretation, and cloud platforms combine these results with laboratory findings and process metadata. Blockchain layers record key compliance events with tamper-evident timestamping, with application dashboards providing access for regulators and supply-chain actors (Dakhia et al., 2025; Liberty et al., 2025; Liu et al., 2025). Embedded readers such as VetStar illustrate the generation of structured screening records, while IoT-enabled packaging provides a broader model for connected food monitoring (Li et al., 2025; Sobhan et al., 2025). Linked batch identifiers should connect analytical results with product movements through processing, distribution and retail, supporting product holds, release decisions and targeted recalls. Confirmed non-compliance should also prompt investigation and corrective action at the source farm (Codex Alimentarius Commission, 2009). Fig. 5 summarizes the integration of an AI/ML-enhanced analytical pipeline feeding into end-to-end digital traceability systems. These layers have different limitations: AI interpretation depends on model generalizability, IoT data exchange depends on device reliability and connectivity, and blockchain auditability depends on trustworthy inputs and governance. Errors can propagate between layers, since an incorrect prediction or misidentified sample can be transmitted and recorded without becoming more reliable.

**Fig. 5:**
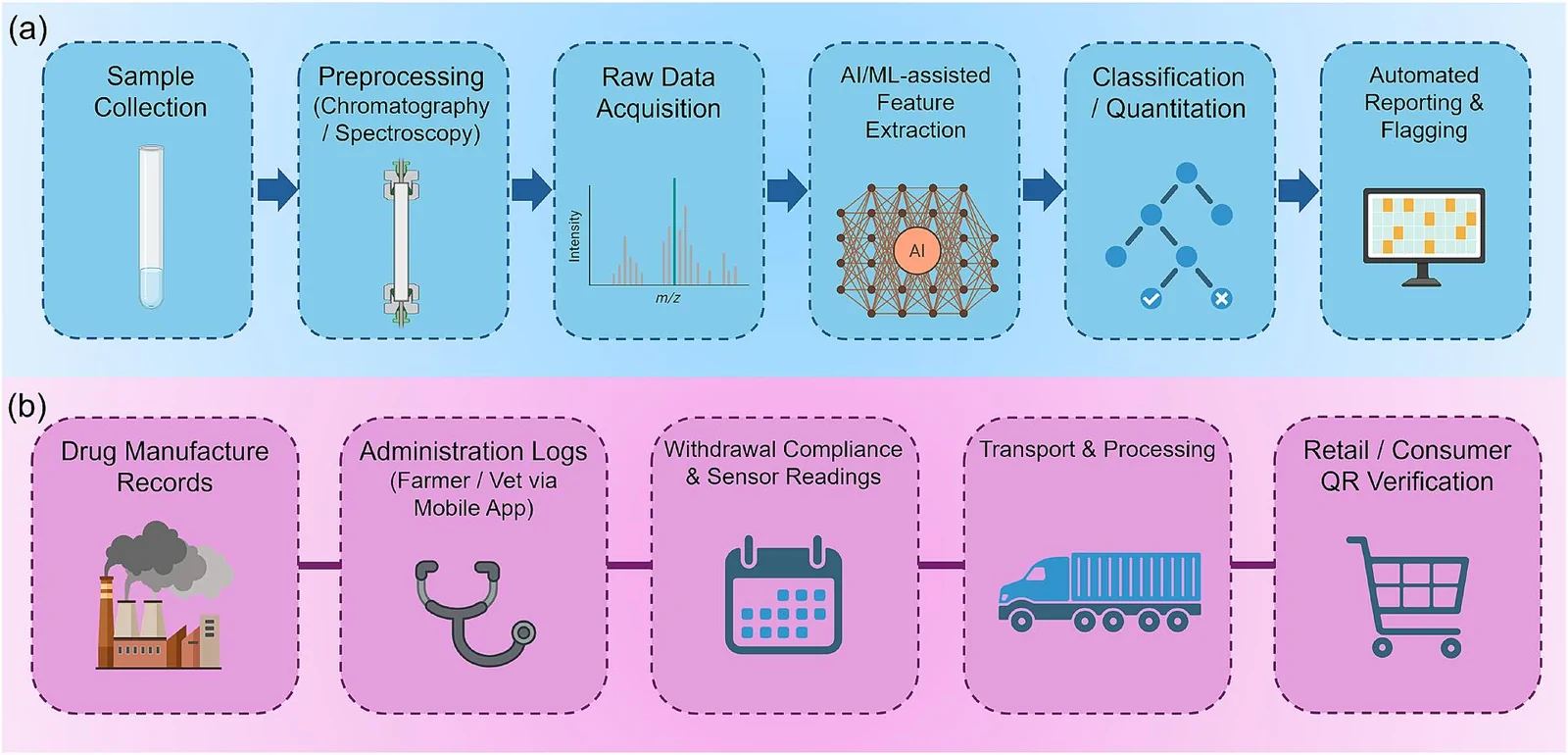
Integrated digital architecture for veterinary drug residue control. (a) Analytical pipeline enabled using AI/ML; (b) End-to-end digital traceability.

Interoperability requires consistent data definitions as well as compatible communication protocols. Shared records should include sample and batch identifiers, species and matrix, analyte or residue definition, result and unit, analytical method, quality-control status and sampling time. Screening results must remain distinguishable from laboratory confirmation. GS1 EPCIS 2.0 supports the exchange of supply-chain events and associated sensor information, but residue-specific vocabularies and validation rules would still be required (GS1, 2025). Each decision should also link to the sampling and custody record, original analytical data, calibration and quality-control results, processing or model version, and the decision rule applied. For example, results expressed per unit volume cannot be compared directly with mass-based limits without an appropriate conversion, and measurements of a parent drug may not represent a regulatory residue definition that also includes metabolites.

Current regulatory reporting provides an operational example of this standardization. European Food Safety Authority (EFSA)'s guidance for the 2026 national control plans for veterinary medicinal product residues specifies an SSD2-compatible data model, controlled terminology and business rules for validating submissions. Its linked tables describe sampling plans and substance–matrix combinations, including analytical methods and relevant performance limits. The framework supports comparison of planned surveillance with reported implementation. A practical direction for connected residue-control systems is therefore to map local laboratory and supply-chain records to established regulatory vocabularies while preserving the distinction between sampling-plan information and individual analytical results (European Food Safety Authority, Salvatore & Vericat Ferrer, 2025).

Cybersecurity must protect devices, communication channels and analytical records. Device authentication, protected data transmission, restricted access and controlled software updates provide a baseline for connected monitoring (Fagan et al., 2020). Additional safeguards should address falsified sample identifiers, compromised laboratory accounts and corrupted model updates, because ledger integrity cannot establish the validity of the original measurement. Governance agreements should distinguish data ownership from access permissions and specify who may submit, correct and reuse records, including their use for model training. They should also assign responsibility for retention, incident response and access to supporting evidence. Sensitive treatment and analytical records could remain in controlled repositories, with shared hashes and audit events supporting verification without exposing the full records.

Realizing this vision requires global alignment. Emerging technologies can exacerbate existing inequalities if deployed only in well-resourced settings; interoperable open standards and capacity building in low- and middle-income countries are therefore prerequisites. Energy-efficient design and data minimization strategies are also necessary to keep the environmental footprint of digital surveillance in check (Sobhan et al., 2025). Ultimately, policy incentives and cross-sector governance frameworks are needed for digital residue-control systems to underpin internationally recognized certifications and support trade and consumer trust (Giannini et al., 2025; Stranieri et al., 2021).

## 5 Sustainable and green analytical approaches

Across farm-level screening, collection-point testing and laboratory confirmation, GAC aims to minimize toxic reagents, waste and energy use while maintaining analytical rigor and regulatory compliance (Gałuszka et al., 2013). The more recent framework of White Analytical Chemistry (WAC) expands GAC by balancing environmental (green), analytical (red) and practical (blue) criteria which should be especially relevant for high throughput residue testing in which operational efficiency is paramount (Nowak et al., 2021). Environmental assessments confirm that analytical laboratories have measurable carbon and water footprints, which shows the need to include energy and emission metrics directly as part of method validation and quality control (Nowak et al., 2023).

### 5.1 Solvent reduction, substitution and separation efficiency

The framework illustrated in Fig. 6 can be assessed through three distinct changes: reducing solvent quantity, substituting solvent identity and improving separation efficiency. Microextraction methods such as SPME, SBSE and DLLME can reduce extraction volumes, but conditioning, desorption and cleaning must remain within the solvent inventory (Armenta et al., 2019). For example, the portable SPME Arrow–MS workflow used 200 μL of acetonitrile for desorption and another 200 μL for washing, while preparation of chicken samples required 20 mL per 5 g of tissue. Miniaturized enrichment therefore did not eliminate substantial upstream solvent use (Lan et al., 2024).

**Fig. 6:**
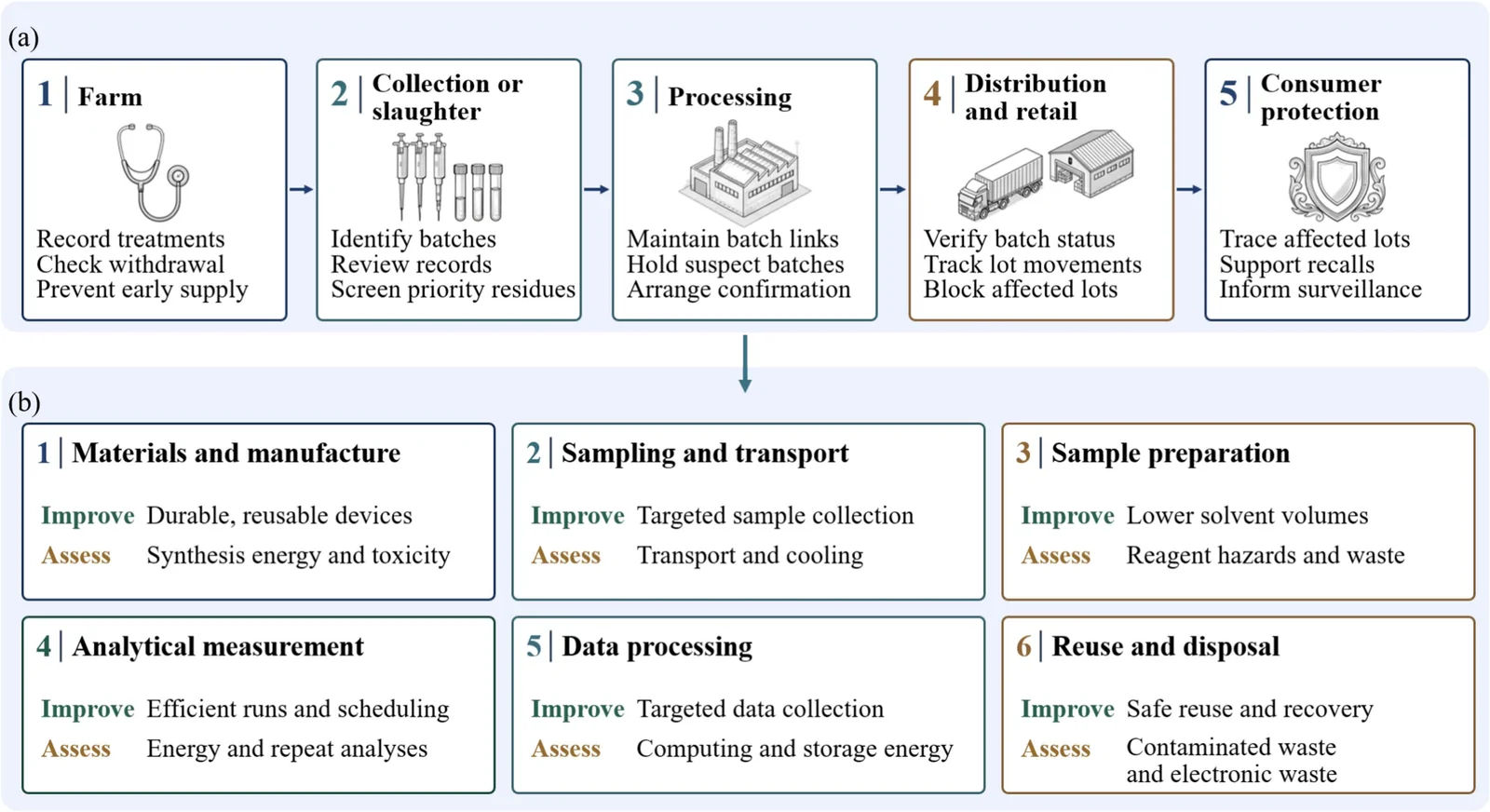
Proposed framework for sustainable farm-to-fork residue control. (a) Control activities from farm treatment records to consumer protection, with analytical results supporting batch decisions and feedback to farms. (b) Potential improvements and environmental burdens across the life cycle of sampling and testing at the relevant control points in Panel A. Environmental benefits require comparative assessment while maintaining analytical reliability.

Solvent substitution requires a separate assessment of analytical suitability and environmental burden. Menthol–octanoic acid DES has been applied to sulfonamide extraction from milk formulas (Shaaban et al., 2023), but low volatility or natural origin does not establish low toxicity (Hayyan et al., 2015). Evaluation should include mixture composition, precursor production, extraction recovery, additional dispersing solvents, biodegradation and the toxicity of degradation products. In a life-cycle assessment (LCA) of a different chemical application, reline showed generally lower impacts than dichloromethane and ethyl acetate but higher impacts than methanol and ethanol. This demonstrates that DES performance depends on the comparator and application (Zaib et al., 2022).

A recent development combines solvent substitution with parallel sample preparation. He et al. coupled a 96-well electromembrane extraction platform, using a 6-methylcoumarin–thymol deep eutectic solvent as the supported liquid membrane, with LC-MS/MS for 22 veterinary drugs in honey, milk and eggs. The optimized extraction lasted 15 min, with reported recoveries of 40–99% and an AGREEprep score of 0.67 (He et al., 2026). This illustrates the potential to combine multi-residue coverage and high-throughput preparation, although the recovery range requires analyte-specific assessment and the greenness score does not establish whole-workflow life-cycle superiority.

Separation changes also redistribute burdens. Lower LC flow rates reduce mobile-phase consumption, but solvent viscosity, heating requirements, ionization compatibility and analytical performance require reassessment (Gaudin, 2025; Napolitano-Tabares et al., 2021). Supercritical fluid chromatography (SFC) can reduce organic solvent demand, although modifiers, make-up solvents, compression energy and CO₂ supply remain relevant (Toribio et al., 2021). Paper-spray MS removes chromatographic mobile-phase consumption, but retains spray solvent, instrument energy demand and matrix-related limitations (Su et al., 2018). The preferred option is consequently the workflow that reduces resource use while maintaining validated residue coverage and decision performance.

### 5.2 Materials, reuse and device lifetime

Material-based strategies include renewable supports, reusable extraction phases and devices with fewer disposable electronic components. Paper microfluidics can reduce reagent volumes and support local screening, but the environmental properties of cellulose cannot be assigned automatically to the complete device. Wax barriers, polymer coatings, recognition reagents and sensing labels must also be considered when evaluating degradation and disposal (Li et al., 2023; Yao et al., 2022). For cellulose-supported metallic sensors, substrate degradation alone does not establish the fate or safety of the remaining nanomaterials (Lospinoso et al., 2025).

Biomass-derived carbon dots and biopolymer composites likewise require assessment beyond feedstock origin. Relevant burdens include heating, chemical modification, purification, washing and material yield. Biocompatibility should be established for the final formulation under relevant exposure conditions, rather than inferred from its biological precursor. Degradation studies should examine released components and their toxicity as well as loss of the original material. These requirements qualify the sustainability claims associated with biomass-derived luminophores and nanocellulose-based electrodes (Korah et al., 2022; Nekoueian et al., 2024; Sanni et al., 2025).

Reusable sorbents distribute manufacturing burdens across multiple analyses only while recovery, carryover and coating stability remain acceptable. Cleaning requirements and replacement frequency therefore belong in the comparison (Lan et al., 2024). Battery-free NFC readouts can avoid disposable batteries, but still require antennas, electronic components and a powered reader (Lazaro et al., 2023). Table 3 summarizes these potential advantages alongside their limitations. Environmental benefit should be assessed per valid analytical result, including failed tests and any required laboratory confirmation.

**Table 3:** Sustainable and green analytical approaches to detect veterinary drug residues.

Approach (green feature)	Representative target(s)	Matrix	Principle / workflow	Reported performance	Potential environmental advantages	Typical limitations	Ref.
Natural DES-DLLME (menthol–octanoic acid DES)	Sulfonamides (7)	Infant/young-child milk formulas	Dispersive liquid–liquid microextraction using a hydrophobic natural deep eutectic solvent; UPLC–MS/MS readout	Low ng·mL^−1^ LODs; validated recoveries within regulatory method criteria (study)	Small extractant volume; total solvent demand includes disperser, sample preparation and LC analysis	DES composition optimization; matrix effects in high-fat dairy	(Shaaban et al., 2023)
μPAD (paper microfluidics) colorimetry	Sulfonamides	Milk	Wax-patterned paper device with colorimetric readout for quantitation	Linear response in milk; minutes-scale analysis (study)	Cellulose substrate; no pumps/electronics; μL reagents; low waste; portable	Susceptible to ambient lighting and sample color; semi-quantitative without calibration	(Sousa et al., 2021)
SBSE (solvent-free enrichment)	Sulfonamide antibacterials (5)	Milk	Stir-bar sorptive extraction; HPLC-DAD determination	Sensitive quantitation; minutes-to-hours extraction with high enrichment	Essentially solventless extraction step; reusable stir bars	Coating selectivity; equilibration time for polar analytes	(Huang et al., 2009)
Green SBSE with ionic-liquid elution	Antiparasitics (macrocyclic lactones)	Milk	SBSE (C18-coated bar) with ionic-liquid elution; HPLC-DAD	Validated recoveries for eprinomectin/doramectin/ivermectin/abamectin	Alternative elution solvent; comparative benefit depends on IL production, quantity and disposal	IL cost/viscosity; need for bar conditioning	(Khalilzadeh Kochameshki et al., 2023)
SPME Arrow (miniaturized, solvent-saving)	Thiabendazole, sulfamethazine, clenbuterol (etc.)	Milk, pork	MOF-nanosheet-coated SPME Arrow; UPLC–QTOF–MS	Selective extraction with HRMS identification (study)	Miniaturized sorptive enrichment; total solvent use depends on preparation, desorption and cleaning	Fiber robustness; per-analyte optimization	(Du et al., 2024)
Portable MS + SPME Arrow (on-site)	Amantadine, thiabendazole, sulfamethazine, clenbuterol, ractopamine	Milk, chicken	SBA-15-SPME Arrow + portable MS with pulsed DC-ESI	Rapid on-site detection; μg·kg^−1^–ng·g^−1^ levels reported (study)	No chromatographic mobile phase; solvent remains necessary for preparation, desorption and washing	Lower resolving power vs. benchtop; limited library coverage	(Lan et al., 2024)
Paper-spray ambient MS (minimal prep)	Multiclass antimicrobials (9)	Beef, chicken, milk, egg	Direct paper-spray MS screening	Identification/quantification without conventional extraction	No chromatographic mobile phase; small spray-solvent volume; MS energy demand remains	Matrix ion suppression; confirmatory testing still needed	(Su et al., 2018)
SFC-MS (CO₂-based chromatography)	Broad classes (including veterinary drugs)	Extracts from animal foods	Supercritical-fluid chromatography coupled to MS	High selectivity/sensitivity; short run times (reviewed)	CO₂-rich mobile phase can reduce organic solvent demand; modifiers and make-up solvents must be included	Modifier choice affects greenness; specialized hardware	(Toribio et al., 2021)
QuEChERS-based multi-residue workflows (optimized for dairy)	≥100 VDRs (example: 103)	Milk & dairy products	Dispersive-SPE cleanup with ACN partition; U(*H*)PLC–MS/MS	Validated recoveries mostly >60%, RSD <20% across 103 drugs	Quick, Easy, Cheap, Effective, Rugged, Safe (reduced solvent/step)	ACN still used; confirmatory standards needed	(Guo et al., 2022)

Environmental advantages describe workflow features and should not be interpreted as demonstrated life cycle superiority unless supported by a comparative assessment. Reported analytical performance remains specific to the cited study conditions.

**Abbreviations:** DES, deep eutectic solvent; DLLME, dispersive liquid-liquid microextraction; UPLC-MS/MS, ultra-performance liquid chromatography-tandem mass spectrometry; μPAD, microfluidic paper-based analytical device; QD, quantum dot; SBSE, stir-bar sorptive extraction; HPLC-DAD, high-performance liquid chromatography with diode-array detection; IL, ionic liquid; SPME, solid-phase microextraction; MOF, metal-organic framework; QTOF-MS (HRMS), quadrupole time-of-flight high-resolution mass spectrometry; DC-ESI, direct-current electrospray ionization; MS, mass spectrometry; SFC-MS, supercritical-fluid chromatography-mass spectrometry; ACN, acetonitrile; QuEChERS, Quick Easy Cheap Effective Rugged Safe; RSD, relative standard deviation; VDR(s), veterinary drug residue(s).

### 5.3 Whole-workflow assessment and digital trade-offs

LCA should compare workflows providing equivalent analyte coverage and decision performance in the same matrix, including consumables, energy, transport, repeat testing and waste treatment. In a study of pesticide extraction from freshwater, Raccary et al. estimated 169 and 331 g CO_2_e per sample preparation for SBSE and SPE, respectively. Vials and caps were major contributors, and electricity supply affected the comparison. These results illustrate the importance of system boundaries but cannot be transferred directly to veterinary residue analysis (Raccary et al., 2022).

Digital tools may support solvent optimization and transport planning, but their additional burdens require explicit accounting. Computational emissions can be estimated from energy consumption and electricity carbon intensity, including facility overhead where applicable (Budennyy et al., 2022). The following annual scenarios are illustrative calculations with assumed inputs, except for the stated device-production proxy. They assume unchanged sampling coverage and analytical performance and are not complete LCAs.

For an AI-assisted laboratory processing 10,000 samples annually, assume a validated solvent substitution reduces consumption from 10 g of conventional solvent to 2 g of replacement solvent per sample. With assumed production-and-disposal factors of 5 and 3 kg CO_2_e/kg solvent, respectively, the annual solvent-related saving is 10,000 × [(0.010 × 5) − (0.002 × 3)] = 440 kg CO_2_e. Assuming 100 kWh for model training and 20 kWh for annual inference, including facility overhead, electricity at 0.4 kg CO_2_e/kWh adds 48 kg CO_2_e. The combined balance is therefore a saving of 392 kg CO_2_e before other changed burdens. Increasing annual training to 1100 kWh reverses this balance to an 8 kg CO_2_e increase. Solvent savings arise from chemical redesign and should only be attributed to AI if demonstrated against the same greener workflow without AI.

For an IoT-supported milk-collection network, assume 100 reusable nodes, each weighing 50 g and lasting three years. Using 1.4 kg CO_2_e per node as a production proxy from a simple IoT device assessment gives an annualized production burden of approximately 47 kg CO_2_e. This proxy is not a measured footprint for a residue sensor (Pirson & Bol, 2021). Assuming 50 kWh annually for operation and data exchange at the same electricity factor adds 20 kg CO_2_e. Avoiding 500 vehicle-km through route consolidation, at an assumed 0.2 kg CO_2_e/km, saves 100 kg CO_2_e, leaving approximately 33 kg CO_2_e of net savings. Retired devices nevertheless generate an annualized 1.7 kg of electronic waste. A one-year device lifetime increases the calculated burden to 160 kg CO_2_e and retired-device mass to 5 kg annually, eliminating the carbon benefit.

These balances remain sensitive to solvent production routes, retraining frequency, device lifetime and actual transport displacement. Additional hardware, replacement batteries and end-of-life treatment can increase burdens. Carbon savings also do not cancel material depletion or ecotoxicity. Deployment should therefore prioritize measured additional savings, reusable devices and reliable analytical decisions.

## 6 Implementation challenges and future perspectives

Despite significant advances in chromatographic, mass-spectrometric, biosensing, and digital technologies for veterinary drug residue control, implementation at scale remains uneven along global animal-source food chains. Moving toward digitally enabled, sustainable residue control requires addressing technical and regulatory barriers; economic and social inequities; and the need to embed residue control within a broader One Health and trade context (Atta et al., 2022; Khalifa et al., 2024; Wu et al., 2023).

### 6.1 Technical and regulatory barriers

The analytical task facing residue-control systems is intrinsically difficult: hundreds of structurally diverse drugs and metabolites have to be quantified at low levels (μg/kg or ng/kg) in fat-rich, protein-dense, or highly processed matrices and often within multi-residue methods. While the use of LC-MS/MS multiclass techniques and HRMS workflows has greatly increased scope, matrix effects, ion suppression and insufficiently generic sample preparation remain persistent causes of uncertainty in routine operation (Wang et al., 2021; Wu et al., 2023; Yang et al., 2025). Non-targeted LC-HRMS introduces further high-dimensional data, in which feature extraction, database coverage and confirmation of tentatively identified compounds pose challenges for standardization and comparability between laboratories (Yang et al., 2025). In parallel, rapid biosensor and nanomaterial-based platforms can provide speed and portability but often have limited robustness, short shelf-life of the biorecognition elements and challenges in providing traceable quantification across complex food matrices. Translating such devices from proof-of-concept studies to accredited methods thus necessitates more systematic evaluation of long-term stability, batch-to-batch reproducibility, ruggedness under field conditions and compatibility with existing confirmatory workflows (Li et al., 2026; Wu et al., 2023; Yang et al., 2025).

Regulatory frameworks provide an extra layer of complexity. MRLs and related decision rules are well developed for a subset of substances and matrices; however, gaps in coverage and inconsistent national standards are very common, which creates challenges for international trade and risk communication (Atta et al., 2022; Okunola et al., 2025). Established validation frameworks support targeted multi-residue methods, as illustrated by the LC-MS/MS validation reported by Gaugain et al. (2025). Non-targeted HRMS, emerging biosensors and AI-assisted interpretation raise additional challenges concerning identification confidence, matrix-specific performance, reproducibility and control of changes to analytical or computational workflows (Atta et al., 2022; Gaugain et al., 2025; Wu et al., 2023; Yang et al., 2025). A shortage of infrastructure, reference materials and proficiency schemes available in low- and middle-income countries frequently means that they are unable to demonstrate compliance with stringent performance criteria, which adds to the dependence on external confirmatory laboratories (Atta et al., 2022; Cannavan and Elliott, 2004).

### 6.2 Economic and social constraints

High-end residue surveillance still is capital and skill intensive. Establishing and maintaining LC-MS/MS or HRMS platforms requires significant investment in instrumentation, climate-controlled facilities, uninterrupted power supply and quality-assured consumables and highly trained analysts (Atta et al., 2022; Wang et al., 2021). A feasible starting model in resource-constrained settings combines validated local screening, scheduled referral to regional confirmatory laboratories and records that remain usable without continuous internet access. Budgets should cover quality control, consumable supply, staff training, maintenance, sample transport, and device replacement. Expansion should depend on demonstrated performance, referral turnaround and cost per valid result, with domestic markets included alongside export chains (Atta et al., 2022; Cannavan and Elliott, 2004). Economic pressures also influence the scope of sampling plans, with limited budgets resulting in prioritization of export-oriented chains while domestic markets are under-monitored. These asymmetries risk entrenching a two-tier food safety system where consumers in high-income importing countries enjoy better control of residue than populations in producing regions (Adegbeye et al., 2024; Atta et al., 2022; Bacanlı & Başaran, 2019).

Social and behavioral factors are equally important. Studies consistently highlight misuse of veterinary drugs, incomplete treatment records, and non-compliance with withdrawal periods, driven by tight liquidity, limited veterinary coverage, and inadequate farmer training (Adegbeye et al., 2024; Mesfin et al., 2024). Short-term market pressures can override residue-control incentives when farmers face disease outbreaks or cash shortfalls. Embedding One Health principles at community level, supported by participatory extension, accessible digital decision-support tools, and targeted communication strategies, has been proposed as a means to realign incentives and co-design practical stewardship approaches (Adegbeye et al., 2024; Mesfin et al., 2024; Qian et al., 2022; Tufa, 2015).

### 6.3 One health integration and global harmonization

Antimicrobial residues, resistance selection and transmission of resistant bacteria are related but distinct concerns. Residues and antimicrobial-resistant bacteria (ARB) can transfer from farms to water bodies, soils, and wildlife, but their detection alone does not establish selection pressure or transmission to humans (Atta et al., 2022; Bacanlı & Başaran, 2019; Khalifa et al., 2024). One Health approaches advocate integrated surveillance that tracks therapeutic use, residue levels, resistance determinants, and environmental contamination simultaneously, enabling more holistic risk assessment and targeted mitigation (Ellahi, Wood and Bekhit, 2023; Khalifa et al., 2024; Qian et al., 2022). An operational starting point is coordinated sentinel sampling that links farm treatment records with residues in edible products, manure and receiving waters, alongside resistant indicator bacteria and selected resistance genes. Shared farm identifiers and sampling dates would support joint veterinary and environmental investigation. Public-health agencies should compare these findings with human AMR surveillance from the same area and period, using compatible bacterial and resistance indicators (WHO, 2021). Such comparisons can identify patterns for follow-up, but co-occurrence alone does not establish transmission or causality. Food-safety authorities should investigate residue non-compliance through product controls and treatment-record review; veterinary and environmental agencies should use resistance and environmental findings to guide stewardship and manure or wastewater management. MRL compliance alone does not establish the absence of resistant bacteria (Atta et al., 2022; Khalifa et al., 2024; Qian et al., 2022).

Global harmonization of standards and data infrastructures is uneven. Okunola et al. concluded that Codex MRLs only cover a minority of drug-product pairs, and that although overt protectionism does not seem to be a major issue across the board, a lot of heterogeneity is introduced where Codex has not been able to set a standard (Okunola et al., 2025). This variability means that compliance costs are higher for exporters, risk communication becomes difficult, and meta-analysis of surveillance data across jurisdictions is impaired (Atta et al., 2022; Okunola et al., 2025). On the technical side, non-targeted HRMS workflows can, in principle, enable international data sharing and retrospective analysis; however, differences in acquisition parameters, spectral libraries and processing pipelines currently limit comparability (Yang et al., 2025). Digital traceability frameworks grounded in blockchain have been suggested as a means of sharing tamper-evident records of veterinary treatments, residue-test results, and product movements across borders, provided data entry, access control and governance are robust potentially supporting both regulatory oversight and private standards (Ellahi, Wood and Bekhit, 2023; Rajput et al., 2025). Systematic reviews of blockchain in food supply chains highlight, however, that interoperability between platforms, data-governance models and long-term sustainability of infrastructures are unresolved challenges that need to be addressed to allow for truly global residue-control architectures (Ellahi, Wood and Bekhit, 2023; Vasileiou et al., 2025).

### 6.4 Phased roadmap for farm-to-fork residue control

Building on existing residue controls, we propose three implementation phases. Their time windows are indicative planning horizons proposed in this review and should be adapted to local capacity. The roadmap should be treated as an adaptable framework rather than a uniform model for all countries. Implementation should align with national responsibilities for oversight, testing and enforcement, while maintaining clear accountability across the supply chain (Codex Alimentarius Commission, 2009). Within available budgets, technology selection should prioritize affordable, sustained operation while preserving adequate coverage, validated analytical performance and access to confirmatory testing (Atta et al., 2022; Cannavan and Elliott, 2004). Greater technological sophistication should justify its additional expense through practical improvements in residue control.

Phase 1, foundations during the first year: regulators and reference laboratories should define priority drug–matrix combinations, sampling points and decision rules. Veterinarians and farmers should maintain treatment records, identify treated animals and verify withdrawal compliance before animals or products enter the food chain. Laboratories should establish validated screening and confirmatory referral procedures. The milestone is a documented pathway linking treatment history, sample identity, analytical results and responsible decision-makers (Codex Alimentarius Commission, 2009).

Phase 2, connected pilots during years 1–3: producer groups, collection centres, slaughterhouses and processors should pilot the workflow in selected supply chains. Screening at collection or processing intake should be linked to batch identification, segregation of suspect products and laboratory confirmation. Laboratory and software teams should connect these records with downstream product movements using the safeguards discussed in **Section 4**. Advancement should depend on demonstrated analytical performance, record completeness and successful tracing exercises. AI-assisted interpretation should enter operational use only after independent validation for the intended matrix and setting.

Phase 3, expansion beyond year 3: supply-chain operators and regulators should extend successful pilots across processing, distribution and retail. Confirmed non-compliance should support targeted withdrawal or recall, consumer notification where appropriate, and investigation of the source farm. Regulators should use findings to update sampling priorities, while veterinarians support corrective treatment practices (van Asselt et al., 2023). Veterinary, public-health and environmental agencies could link residue findings with antimicrobial-use and resistance surveillance through the governance arrangements described in Section 6.3.

Across all phases, program evaluation should track withdrawal-record completeness, screening false-negative performance, confirmation turnaround, and the proportion of implicated lots located during tracing exercises. Cost, solvent consumption, waste and energy per sample should be assessed across screening and confirmation so that expansion remains affordable and environmentally justified (Nowak et al., 2023).

## 7 Conclusion

Effective residue control requires reliable measurements linked to timely action. A farm-to-fork system should connect prevention during treatment and withdrawal with screening at collection or slaughter, laboratory confirmation, traceable product control through retail, and corrective action at the source farm. Rapid biosensors can support this sequence when validated for the intended drug and matrix. Digital tools can connect analytical results with treatment and batch records, but their value depends on data quality, interoperability and accountable decisions. Environmental benefits require assessment of the complete testing workflow, including digital energy use and electronic waste. The phased roadmap prioritizes validated screening and confirmation before wider digital integration, with implementation adapted to local capacity. Linking residue surveillance with antimicrobial-use, resistance and environmental data can support One Health action, while preserving the distinction between residue compliance and AMR risk.

## Author contribution statement

All authors significantly contributed to the conception and design, data acquisition, or data analysis and interpretation; participated in drafting the article for its essential content; and consented to be responsible for all facets of the work.

## CRediT authorship contribution statement

**Marzieh Ramezani Farani:** Writing – original draft, Supervision, Conceptualization. **Danial Mirzaee:** Writing – original draft, Visualization. **Anahita Voosough:** Writing – original draft. **Afrooz Hatami:** Writing – original draft. **Kong Chanmey:** Writing – original draft. **Seyed-Hassan Miraei Ashtiani:** Writing – review & editing. **Aliya Sheik:** Writing – original draft. **Iraj Alipourfard:** Writing – original draft. **Alexandros Ch. Stratakos:** Writing – review & editing, Validation. **Sergey A. Piletsky:** Writing – review & editing. **Seung-Kyu Hwang:** Writing – review & editing, Visualization. **Yun Suk Huh:** Writing – review & editing, Validation, Supervision, Methodology, Funding acquisition, Conceptualization.

## Declaration of competing interest

The authors declare that they have no known competing financial interests or personal relationships that could have appeared to influence the work reported in this paper.

## Data Availability

Data will be made available on request.
